# Enhancing the User Experience of a Perioperative Digital Health Tool for Information Exchange Using a Human-Centered Design Thinking Approach: Qualitative Observational Study

**DOI:** 10.2196/79349

**Published:** 2026-01-12

**Authors:** Charlé Steyl, Carljohan Orre, Greg Foster, Hanel Duvenage, Michelle S Chew, Hyla Louise Kluyts

**Affiliations:** 1 Department of Anaesthesiology, School of Medicine, Sefako Makgatho Health Sciences University, Pretoria, South Africa; 2 Department of Computer Science and Media Technology, Malmö University, Nordenskiöldsgatan 1, Malmö, 211 19, Sweden; 3 Department of Information Systems, Rhodes University, Makhanda, South Africa; 4 Safe Surgery South Africa, Johannesburg, South Africa; 5 Department of Anaesthesiology and Intensive Care, Linköping University, Linkoping, Sweden; 6 Department of Perioperative Medicine and Intensive Care, Karolinska University Hospital, Stockholm, Sweden

**Keywords:** patient-reported outcome measures, person-centered care, patient participation, digital health, perioperative care, user-centered design, user experience research, human-centered design, universal design, design thinking

## Abstract

**Background:**

Perioperative patient-reported outcomes (PROs) allow patients to share their experiences of surgical procedures with their health care teams using standardized measures. Despite increasing recognition of their value, PROs are not routinely used in clinical practice, partly due to limited evidence of their impact on traditional clinical outcomes and uncertainty among clinicians about their use. Digital health tools offer a promising way to integrate PROs into clinical workflows and enhance patient-clinician interaction, but their success depends on person-centered design to ensure usability and relevance. Safe Surgery South Africa, a nonprofit organization, developed the Perioperative Shared Health Record (PSHR), a secure web-based tool that enables patients to share personal health information and PROs with their anesthetist and surgeon before and after surgery. Initial implementation revealed significant user experience challenges, which contributed to poor uptake.

**Objective:**

This study aimed to explore factors influencing the PSHR user experience in a low- and middle-income country (LMIC) using human-centered design principles.

**Methods:**

This observational qualitative user experience study followed the 5 design thinking stages: empathize, define, ideate, prototype, and test. Semistructured interviews were conducted with postoperative patients from both the public and private health care sectors, including those with and with no prior experience using the PSHR. Thematic analysis followed the 6-phase framework described by Braun and Clarke and was structured using Karagianni’s Optimized Honeycomb user experience model. A problem statement was developed, followed by ideation to explore solutions. Paper prototypes were created, refined, and tested through observation, interviews, and validated usability questionnaires.

**Results:**

In the *empathize* stage, 22 interviews were conducted in the private and public health care sectors in South Africa; 7 participants had previous experience using the PSHR. In the *define* stage, participants emphasized the need for connection, feedback, information, and support through their surgical journey. Contrary to expectations, patients were not discouraged by the length of questionnaires if they perceived them as purposeful. In the *ideate* stage, the team considered user expectations and PSHR integration into care processes. In the *prototype* stage, low-fidelity mock-ups were created and refined into paper prototypes. In the *test* stage, testing with 5 participants highlighted the importance of trust, communication, and user-friendly interfaces. Feedback loops and clinician engagement were identified as key motivators for sustained use. The mean usability questionnaire scores indicated excellent usability and high levels of user satisfaction across most domains.

**Conclusions:**

This study is one of the first to apply human-centered design principles to a perioperative digital health tool in an LMIC setting, addressing usability challenges and patient engagement. Key user experience factors influencing patient engagement included communication, feedback, and access to information throughout the surgical journey. Digital health tools such as the PSHR can strengthen communication and support person-centered perioperative care by integrating PROs into clinical workflows and care processes.

## Introduction

Patients presenting for surgical procedures often feel vulnerable and may become overwhelmed by information that lies outside their usual frame of reference. Many also experience significant physical and emotional symptoms before and after their operation [1,2]. Time constraints and brief interactions during busy ward rounds can limit opportunities for patients to voice concerns or seek clarification [1,3]. In this context, there is a risk that patients feel depersonalized: reduced to passive participants in a system rather than active participants in their own treatment [1]. Furthermore, perioperative clinicians such as surgeons and anesthesiologists often prioritize traditional problem-focused postoperative outcomes such as morbidity and mortality rates, which do not necessarily reflect the outcomes that matter most to individual patients [4-6].

Person-centered care addresses these challenges by recognizing the individual behind the patient: a human being with values, emotions, and goals, and by fostering a partnership that supports patient autonomy and active participation in care decisions [7,8]. Evidence suggests that patients who are empowered and engaged in their own health care may have better outcomes [3,9-11]. Perioperative patient-reported outcomes (PROs) provide one means of achieving this by allowing patients to communicate their experiences of surgical procedures using standardized questionnaires, known as patient-reported outcome and experience measures (PROMs and PREMs) [2,4,12-14]. Various PROs have been defined in the perioperative sphere, including patient satisfaction, quality of recovery (a short-term outcome after surgery), and quality of life (a longer-term outcome after surgery) [14]. Perioperative PROMs and PREMs give individual patients a means to communicate how they are recovering after surgery in such a way that it can be compared between patient groups and procedure types [5,14]. The data can be used to track quality of care over time [6].

Consensus guidelines recommend the use of PROMs and PREMs in clinical research, and their implementation in perioperative care is increasingly studied [2,6,15-18]. In daily practice, however, the routine use of PROs is hampered by their time-intensive nature, limited evidence linking them to traditional outcomes such as complications or mortality, and uncertainty among clinicians and patients regarding their value [2,6,12,16,17]. These challenges may reflect insufficient person-centeredness in the application of PROs and a lack of responsiveness of health care teams to the information provided by patients [2,19].

Digital health tools offer a promising way to integrate PROs into clinical workflows and to enhance communication between patients and clinicians [17,18,20]. Achieving this, however, requires a human-centered design approach to ensure that digital tools are responsive to diverse user needs and care contexts [21]. Human-centered design forms part of the broader design thinking framework: an empathetic, iterative process that involves end users throughout development to create tools that are understandable, useful, and enjoyable to use [22-25]. Applying these principles through user experience research, which uses interviews, surveys, and usability testing to explore how people interact with digital systems, helps developers create tools that are more intuitive, engaging, and relevant to real-world care [21,26]. Learning from established digital health platforms and implementation of electronic medical record systems can help create digital health tools that support care across the patient journey [27-31].

Perioperative digital health tools have shown promise in high-income countries (HICs) [17,18], but their implementation and use remain underexplored in low- and middle-income countries (LMICs). In South Africa, barriers to large-scale adoption of digital health solutions include limited digital literacy and unequal access to technology and internet across social, economic, and geographic groups [32,33]. These disparities reflect broader inequalities within the health system, where a tax-funded public sector provides care for most of the population but is underresourced, while a private sector funded through medical schemes and out-of-pocket payments serves a minority yet absorbs a large share of resources [34-36]. The public sector continues to rely largely on paper-based documentation, with uneven implementation of systems to capture routine health information and limited electronic record keeping compared with the private sector [37-41]. The private sector is data-rich, with more electronic data systems, but its datasets are typically siloed and not routinely accessible to public governance systems, clinicians, or patients [39,42]. Neither sector currently supports routine or large-scale capture of PROs, limiting opportunities to measure and improve perioperative care from the patient perspective. Addressing these challenges requires context-specific digital solutions that can be designed to strengthen perioperative care in South Africa.

**Figure 1.**
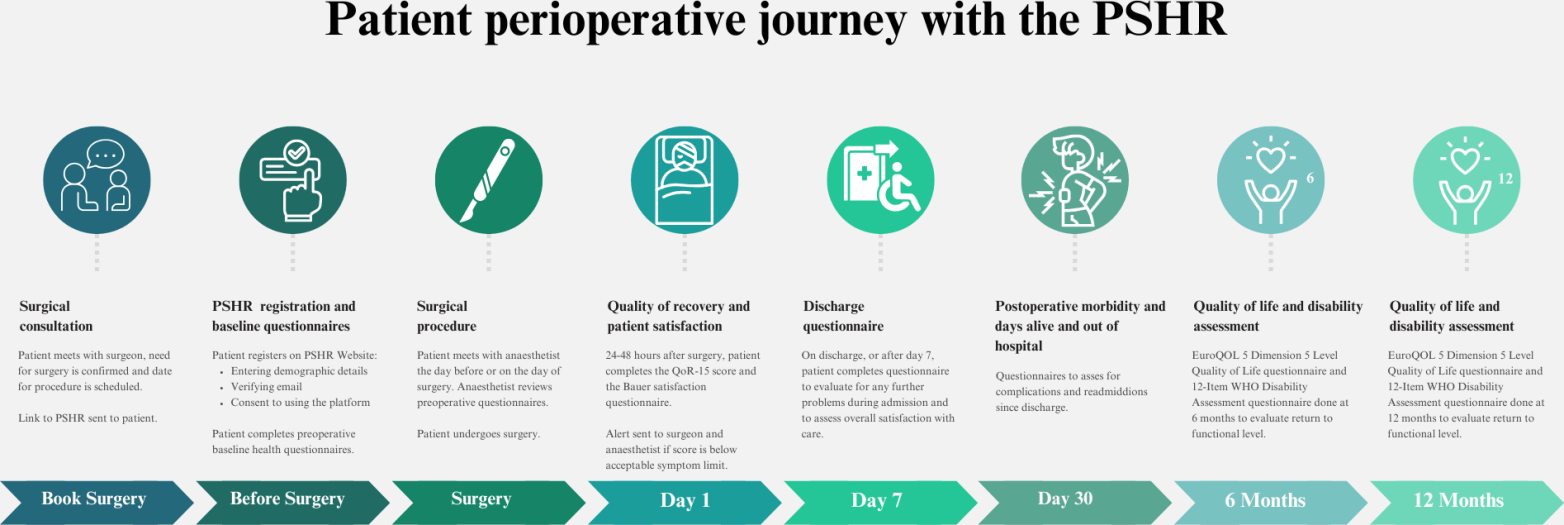
Patient journey when using the PSHR. EuroQOL: European Quality of Life questionnaire; PSHR: Perioperative Shared Health Record; QoR-15: 15-Item Quality-of-Recovery questionnaire; WHO: World Health Organization.

In response to these challenges, Safe Surgery South Africa [43], a research-driven nonprofit organization, developed the Perioperative Shared Health Record (PSHR) [44], a web-based digital tool enabling patients to share baseline preoperative data and postoperative PROs with their surgeon and their anesthetist for up to a year after surgery. Preoperative data can be used in risk stratification and shared decision-making, whereas postoperative PRO data can improve patient care. Data are stored on a secure server but are accessible to both patient and clinician. The system was designed to function across the public and private health care sectors to promote broader accessibility. Figure 1 describes the use of the PSHR in capturing the perioperative journey of a surgical patient.

Initial use of the PSHR in the private health care sector, during the South African Collaborative Surgical Outcomes Study (SACSOS; ClinicalTrials.gov NCT05052021), identified numerous user experience challenges that led to low patient and clinician engagement, which reduced the effectiveness of the PSHR. The registration process was cumbersome, requiring active support for patient users to complete it. In addition, some questionnaires were perceived as lengthy and burdensome, potentially discouraging patients from completing subsequent assessments. As many of the questionnaires are standardized tools designed for specific purposes, content modifications were not always feasible.

The aim of this observational study was to determine the factors that influence the patient user experience of the PSHR as a tool to support perioperative care. The primary objective was to evaluate the user experience of patients in South Africa who had previously used the PSHR during SACSOS. Further objectives were to explore the user needs of patients who have no prior experience with the PSHR and to gain deeper insights into the future design requirements of the PSHR.

To achieve this aim, this study used a human-centered design thinking approach, which required a multidisciplinary team that could combine clinical insight, technical expertise, and practical experience. In this study, anesthesiologists contributed their understanding of perioperative workflows and patient-clinician communication. The information systems, computer science, and media technology researchers applied user experience and design thinking principles to translate patient needs into feasible design solutions. One of the anesthesiologists (CS), with research expertise in PROs, and one of the information systems researchers (CJO), with personal experience as both a patient and a hospital representative, brought perspectives that ensured that the patient remained central throughout the course of the project. The researchers brought together expertise from South Africa and Sweden, combining experience with emerging and advanced digital health systems and perspectives from LMIC and HIC settings. These efforts aim to improve the PSHR’s usability and provide insights for more person-centered digital health design.

## Methods

### Ethical Considerations

This observational qualitative user experience study was approved by the Sefako Makgatho Health Sciences University Research Ethics Committee on November 16, 2023 (SMUREC/M/513/2023:IR), and registered on the National Health Research Database on January 28, 2025 (NHRD: GP_202501_070). Written consent was obtained from all participants and patient privacy and confidentiality was respected by deidentifying patient data and removing any identifiable features from images used in publication. Participation was voluntary with no compensation paid to participants.

### Setting

The study took place in South Africa, with postoperative patients and carers recruited via purposeful sampling in both the private (insurance-funded) and public (tax-funded) health care sectors. These 2 sectors are vastly different in South Africa, with a different patient demographic and a significant difference in availability of resources.

### Research Team

The research team consisted of 4 female researchers and 2 male researchers. Three of the female researchers (CS, HK, and MC) are practicing anesthesiologists. HD has a research and development background. HK is the founder of Safe Surgery South Africa and focuses on data-driven solutions to improve perioperative risk stratification and surgical outcomes. CS has a clinical and research interest in PROs and previously recruited patients for the PSHR as part of the SACSOS study, maintaining professional relationships with these participants. One of the male researchers, CJO, comes from a digital health and media background and is a patient representative on a hospital management board after surviving cancer. CJO is also involved in an online cancer rehabilitation program spearheading the use of PROMs and PREMs to improve care processes. The other male researcher, GF, comes from a user experience and design science research background.

### Methodology

This study was informed by human-centered design principles. The design thinking process was used to structure the study around 5 phases: empathize, define, ideate, prototype, and test [23,25]. In keeping with a person-centered approach, participants informed the project from the outset by sharing their perioperative experiences and needs. These insights guided ideation and design decisions by the investigators, and participants were reengaged during prototype testing to evaluate and improve solutions based on earlier input.

#### Phase 1: Empathize

The first phase of the project focused on empathizing with PSHR users by interviewing 2 distinct groups. The first group, recruited from the private health care sector, had prior experience using the PSHR before and after surgery through SACSOS (group 1: PSHR experience). The second group included individuals from both the public and private sectors who had undergone surgery but who had no prior exposure to the PSHR (group 2: no PSHR experience). Participants were invited to take part via email, telephone, or by in-person invitation.

For ease of reference when presenting participant quotations, each participant is assigned a letter prefix. “P” denotes participants in group 1, who had used the PSHR before, “U” denotes participants in group 2 from the public sector, and “I” denotes participants in group 2 from the private sector.

Semistructured interviews were conducted between November 2023 and January 2024. Through storytelling, empathy maps and patient journey maps were created. Demographic data were recorded in REDCap (Research Electronic Data Capture) [45] and exported to MS Excel (version 2411; Microsoft Corp). The interviews began by exploring all patients’ perioperative experiences. Group 1 participants were then asked about their experiences using the PSHR, while group 2 participants received a brief demonstration of the PSHR before discussing their expectations of a digital tool for perioperative information exchange. The interview guide is included in Multimedia Appendix 1.

Interviews were recorded and transcribed using transcription software (Transcribe—Speech to Text, version 4.20.5; DENIVIP Group LLC) on an iPad dedicated to the project. Transcriptions and audiovisual files were stored in a password-protected online folder. Transcriptions were checked for accuracy by CS and HD and reviewed by all investigators before data analysis. Transcriptions of the initial interviews were systematically coded and analyzed thematically using Nvivo (release 14.23.4(49); Lumiere) by CS and HD. The thematic analysis followed the 6-phase framework described by Braun and Clarke [46]. To explore the main themes related to participants’ experiences and expectations of the PSHR, responses were systematically coded and categorized using Karagianni’s Optimized Honeycomb model [47]. This model, commonly used in user experience research, structures the analysis of how users interact with a product by breaking down their experiences into 3 primary dimensions: Use, Feel, and Think [47-51]. By applying this framework, we identified patterns in the data and gained deeper insight into the factors influencing user experience of the PSHR.

#### Phase 2: Define

The information obtained during phase 1 was used to create a problem statement and summary of findings.

#### Phase 3: Ideate

Insights from the initial interviews and the defining phase informed an ideation phase, during which various solutions were brainstormed by CS, HD, HK, CJO, and GF to enhance future implementation of the PSHR and also taking into account the interoperability with electronic health records.

#### Phase 4: Prototype

Paper prototypes for the PSHR were created in Balsamiq Wireframes for Desktop (version 4.8.1; Balsamiq Studios LLC). Paper prototypes were refined based on research team group discussions and during user testing.

#### Phase 5: Test

User testing with the paper prototypes took place in December 2024 with patients and carers who were recruited via email, telephonically, and in person, with the aim to recruit both patients and carers who had used the PSHR before (“expert users”) to determine whether insights learned from them during initial interviews had improved their user experience. and patients and carers who had no prior experience of the PSHR (“novice users”), to determine their first time user experience with the system.

As all the expert users would be from the private health care sector, novice users were recruited from the public health care sector. To recruit the expert users, attempts were made to contact all 7 participants from group 1; 3 could not be reached at all, 1 initially agreed but later withdrew, and 3 consented and participated. For the novice users, we intentionally sought individuals with no prior exposure to the platform, including through earlier interview phases, to ensure unbiased, first-time user perspectives. This necessitated recruitment of new participants. Eligibility for user testing included being conversant in English, having basic familiarity with mobile phone and computer use and with the use of the internet. Testing was undertaken by 4 investigators (CJO, CS, GF, and HD), 1 acting as the “computer” to change paper “screens” based on user actions, 1 facilitating the scenario, and 2 observing the interaction; sessions were also audio-recorded for later analysis. For ease of reference, “T” denotes participant responses in the user testing phase.

Participants were asked to complete four tasks during the prototype testing: (1) registering and consenting, (2) completing preoperative baseline questionnaires, (3) finding additional information on the PSHR, and (4) completing postoperative quality of recovery and patient satisfaction questionnaires (Multimedia Appendix 2).

The System Usability Scale (SUS) was used to assess usability after the user prototype testing, as this is a well-established tool that has been found to have good reliability to evaluate the usability of digital systems. The SUS is a 10-statement scale for usability of electronic health applications with good reliability (Cronbach α=0.911) and good face validity [52]. The SUS score ranges from 0 to 100 where higher scores indicate greater usability [53]. A mean SUS score of 68 (SD 12.5) represents the average benchmark for digital health apps [54].

User experience and usability aspects were assessed after prototype testing with the User Experience Questionnaire (UEQ), a well-established tool that evaluates 6 aspects including attractiveness, effectiveness, perspicuity, dependability, stimulation, and novelty with 26 pairs of terms that are scored from 1 to 7 [55]. The questionnaire has good construct validity and good reliability (Cronbach α for 5 of the 6 aspects is above 0.7) [55]. Scoring is done with a downloadable tool, with values ranging between −3 (horribly bad) and +3 (extremely good) [55]. Scores should be evaluated against current benchmarks, freely available for download [56,57].

Prototype user testing was analyzed by 4 investigators (CJO, CS, GF, and HD) who took part in the process using interviews and observation. Thematic analysis of the user tests was done based on research team discussions following the user tests. The SUS and the UEQ were scored in MS Excel according to the guidelines in their reference papers [53-55,57].

## Results

### Phase 1: Empathize

A total of 22 initial semistructured interviews were conducted as part of empathizing with users. Participant demographics are summarized in (Table 1). All the participants had access to a mobile phone; all but 2 participants in group 2 in the public sector had access to the internet on their mobile phone. Most participants (16/22, 73%) usually used their mobile phones for accessing the internet, whereas 4 out of 22 (18%) participants preferred to use a computer for internet access, and 2 out of 22 (9%) participants did not use the internet at all.

**Table 1.** Demographic data for semistructured interviews conducted for phase 1: empathize.

ID^a^	Age (years)	Sex	Language	Race	Prior PSHR^b^ experience	Education	Surgery
P1	33	Female	English	Black	Yes	After school qualification	Major abdominal
P2	42	Female	English	White	Yes	After school qualification	Major abdominal
P3	62	Female	Afrikaans	White	Yes	After school qualification	Major abdominal
P4	22	Female	English	Black	Yes	After school qualification	Major abdominal
P5	80	Male	Afrikaans	White	Yes	After school qualification	Major abdominal
P6	72	Male	Afrikaans	White	Yes	After school qualification	Major abdominal
P7	46	Male	Afrikaans	White	Yes	After school qualification	N/A^c^—assisted family member
U1	34	Female	Setswana	Black	No	Secondary school not completed	Major abdominal
U2	22	Male	Northern Sotho	Black	No	Secondary school not completed	Major abdominal
U3	43	Female	Tsonga	Black	No	Secondary school completed	Vascular surgery
U4	65	Male	Setswana	Black	No	Secondary school not completed	Vascular surgery
U5	48	Female	Afrikaans	White	No	Secondary school not completed	Bariatric surgery
U6	29	Female	Northern Sotho	Black	No	After school qualification	Bariatric surgery
U7	42	Female	Afrikaans	Colored^d^	No	After school qualification	Bariatric surgery
U8	39	Female	English	Black	No	After school qualification	Bariatric surgery
U9	42	Male	French	Black	No	Secondary school completed	Vascular surgery
U10	45	Female	Setswana	Black	No	After school qualification	Vascular surgery
I1	64	Female	Afrikaans	White	No	After school qualification	Orthopedic
I2	41	Female	Afrikaans	White	No	After school qualification	Bariatric surgery
I3	54	Female	Afrikaans	White	No	After school qualification	Breast surgery
I4	76	Male	Afrikaans	White	No	After school qualification	Orthopedic
I5	42	Male	Setswana	Black	No	After school qualification	Orthopedic

a User ID explanation: “P” denotes participants who had used the PSHR before, “U“ denotes participants from the public sector with no prior experience of the PSHR, and “I” denotes participants from the private sector with no prior experience of the PSHR.

b PSHR: Perioperative Shared Health Record.

c N/A: not applicable.

d In South Africa, the term “Colored” refers to a distinct cultural and ethnic group with mixed ancestry, recognized as a separate demographic category.

Thematic analysis of the interviews identified 3 main themes: Patient Journey (both groups), PSHR Experience (group 1), and PSHR Expectations (group 2), each with subthemes related to patient engagement and user experience. Detailed findings are provided in Multimedia Appendix 3.

#### Patient Journey

In understanding the patient perioperative journey (main theme), the following subthemes were identified in both user groups: information-seeking behaviors, emotional response, postoperative difficulties, interaction with health care providers, and advice to other patients. Some participants actively sought more information, either by doing online searches or talking to family members or patients who have been through a similar situation.

*I was checking [online] how long it’s going to be the operation. Okay. Yeah. And how was going to be the pain? How I was cut, a lot of it*.

[U1]

*I go and check like, like the food I have to eat. And then the thing I didn't Google about it is the pills the most. But you know that if you want to go look, you can go, you can go find*.

[U3]

*So I had my sister-in-law, who is a general practitioner, check for the results and then she was the one [that told me]*.

[P2]

*And I was also following [on social media], uh, people that will talk about their experience, you know*.

[P1]

*I must say the information from other patients helped a lot, knowing what someone else went through, their experiences, how they felt, what the cost implications were, how they paid it, all of that helped a lot*.

[I2]

In both the private and public sectors, there were participants who indicated that they avoided looking for any additional information:

*I don’t really want to Google stuff because you always, there’s always stuff. Too much information*.

[P3]

*I think that would've scared me off a little bit more if I knew truly what was to come*.

[P4]

*...because you know when you Google things you don’t always get the right information. And it can be very scary*.

[I3]

*So, I give up to an extent that I did not even want to stress myself about the Google information. Because others there are just making some speculations*.

[U10]

All the participants experienced some form of emotional turmoil in the time after their diagnosis and before they had surgery, with some describing being in denial, feeling helpless, and isolated:

*I started like shaking and getting worried. Yes. Like now it’s getting worse. And like I took it easy, like okay, fine. I went back to work instead of going to the doctor*.

[I5]

*But now it all became too much. It just felt like you take one step forward and like five steps back… It felt like I was in constant pain and I also felt very helpless*.

[P1]

*It was very... because nobody can come in with you and then you're there alone and then they don't communicate well, doctors all the time, some of them*.

[P2]

One participant said an uplifting conversation with her surgeon gave her hope before her surgery and this helped her carry on with her treatment:

*That answer, that one sentence, and with such conviction, uh, brought back my, um, my hope*.

[P3]

Participants in both groups described some physical difficulties in the postoperative period:

*I think the first two weeks were the hardest really. And the vomiting was much worse at home. Yeah. Uh, the pain also from eating was really terrible*.

[P4]

*But that was also the worst thing that I had the operation, because it was very painful! I didn’t expect it would be so painful!*

[U7]

Two participants commented that being informed and being able to contact their surgeon made the perioperative journey easier.

*So being informed. Yeah. Makes you feel more reassured*.

[P3]

*The interesting thing about this surgeon’s practice, that I have not come across before, is that he gives you a 24hr whatsapp number that you can use any time of the day if you have problems or questions. There is always someone that responds—that is not something that everyone would do*.

[I4]

Participants in both the public and private sectors described their interaction with their health care providers in positive terms, and they valued in-person communication:

*It made such a difference that [the anaesthetist] were there and [she] could explain to us what was going to happen, it made us feel a lot more secure and calm*.

[P6]

*...what I felt was more these people are taking care of me. I was positive. I could see these other people (points to other patients in ward), they are getting more healthy*.

[U2]

Participants offered advice to others that reflected both practical and emotional preparation for surgery. Some emphasized the need to prepare physically by doing breathing exercises and maintaining mobility, while others recognized the emotional impact of surgery on the patient and their families.

*It’s the emotional side of things that takes quite a toll. But not only on [the patient], but on [the family] too. So I think the emotional strain on both was tough*.

[P7]

Several advised future patients to listen to their doctors, follow instructions carefully, and trust the care team. Others highlighted the importance of patience and realistic expectations, especially regarding the time needed for healing:

*I would tell them that it is very important to listen to what the doctor tells you. To stick to the rules...And I would tell them that they shouldn’t be scared to go through with it*.

[I1]

*I would explain my journey the way it is, then they can come here and get that help because it’s a better help than any other*.

[U3]

*As a patient, I will say first thing first you need to be patient. You need like...healing is a mercy. It won't just happen overnight*.

[I5]

#### PSHR Experience

Group 1 included 6 patients and 1 family member who had used the PSHR. Their user experience, analyzed according to the Optimized Honeycomb model, is summarized in (Table 2), with supporting quotations in Multimedia Appendix 3.

**Table 2.** Codes related to experience or expectations of the Perioperative Shared Health Record^a^.

	Subthemes^b^^,^^c^						
	Use			Feel		Think	
	Findable	Accessible	Usable	Desirable	Credible^d^	Useful	Valuable
PSHR^e^ experience: Group 1: PSHR exposed; private sector; 7 interviews conducted with 4 women and 3 men (2 Black and 5 White, aged 18‐80 years)	WhatsApp link preferred (9) Email not used frequently (5) Desktop used initially (1)	Mobile phone access preferred (6) Device limitation (2) Postoperation no access to glasses (1) Not comfortable with technology (1)	Easy to use (4) Technical problems/bugs (4) Login process difficult (2) Importance of feedback (2) Font size too small (1) Medical language tricky (1) Loss of interest over time (1)	Can complete at home (2)	No codes	Personal connection to doctor (10) Means to feedback from doctor (8) Patients’ ability to express their needs (5) Benchmarking (4) Ability to give family access to platform (1)	Improved care (5) Patient involvement (1) Altruism (1)
PSHR expectations: group 2: PSHR unexposed; public sector; 10 Interviews conducted with 7 women and 3 men (7 Black, 2 White, 1 Colored^f^ , aged 22‐65 years)	No codes	WhatsApp link preferred (12) Device limitation (4) Mobile phone access preferred (3) Email not used frequently (3) No access to phone postoperatively (2)	No codes	No codes	No codes	Communication channel (9) Means to feedback (5) Benchmarking (4) Efficiency (3)	Improved care (2) Personal connection (2)
PSHR expectations: group 2: PSHR unexposed; private sector; 5 interviews conducted with 3 women and 2 men (1 Black and 4 White, aged 41‐76 years)	No codes	WhatsApp or SMS (2) Email not easily accessible (2) Email link useful (1) No access to phone postoperatively (1)	No codes	No codes	Negative feedback may impact care (1)	Efficiency (3) Source of information (3) Curated list of information (1) Information about doctor (1) Communication channel (1) Link to other patients/support groups (1)	Altruism (6)

a Main theme: PSHR Experience (group 1) and PSHR Expectations (group 2).

b Codes from the text were sorted according to the 7 aspects of user experience from the Honeycomb Model (Findable, Accessible, Usable, Desirable, Credible, Useful, and Valuable) [47]. Code names are included under each aspect, with the code count in parentheses.

c Subthemes: Use, Feel, and Think according to Karagiani’s Optimized Honeycomb Model [47].

d Note that the aspect Credible falls under both Feel and Think.

e PSHR: Perioperative Shared Health Record.

f In South Africa, the term “Colored” refers to a distinct cultural and ethnic group with mixed ancestry, recognized as a separate demographic category.

Most participants preferred to follow WhatsApp links to find their way to the PSHR on their smartphones:

*I do prefer that it was easy to use on my phone. So if it can be improved, it must still just be improved. Mainly for, for like a smartphone*.

[P1]

*Yes, because on personal email you're not visiting that often, so it gets lost with all the other stuff. So, I believe your preferred communication is WhatsApp*.

[P7],

One participant commented on the potential difficulty of using a smartphone on the first day postoperatively:

*It was a bit difficult. Different. If you wear glasses and you don't have glasses on, and you're on morphine. But it wasn't, it wasn't impossible*.

[P2]

One of the older participants indicated that they were not comfortable with technology, which is a potential barrier to using a digital tool such as the PSHR:

*No, I’m not so comfortable with my phone. The internet on there is not something that I usually use*.

[P5]

Contrary to expectations, the length of the questionnaires was not perceived in a negative light:

*It did just go on and on and on. Um, I, I think in my head it was just all part of, just part of the process, preparation and the process you had to do, you know, and making sure that everything is fine*.

[P1]

*It was easy to answer. It doesn't take too long*.

[P4].

However, there was some concern about medical jargon and font size:

*There’s some, um, uh, of the wordings and stuff that I really didn't understand*.

[P3]

*I think the only complaint if I need to complain about improvements will be the size of the font perhaps*.

[P7]

Participants valued the PSHR for enhancing their engagement and improving the quality of care they received:

*...[the doctor] was able to quickly know and come back and improve my care, you know?*

[P1]

*You feel that you were more involved in the planning of your care*.

[P2]

Feedback from the surgeon or anesthetist emerged as an important motivator for continued use:

*[The anaesthetist] had read what was going on. She came and she asked what was going on, and I explained that and she worked around it and talked to the staff*.

[P1]

*If I didn’t get feedback, I wouldn't have filled in anymore. I would've done the first one and left it at that*.

[P2]

Participants appreciated being able to reflect on their recovery:

*All of this is quite relevant because it lets you think about your own wellbeing and progress*.

[P6]

Interestingly, some participants were also motivated by altruism, expressing a desire to help others:

*...if my information can help somebody else get through a very difficult situation...then I feel it’s worth it.*

[P3]

Suggestions for improving the PSHR centered around information sharing and being able to contact patients who had been through a similar procedure:

*I suppose especially for, for large operations, it might help people to know who the anaesthetist is and have like a name and a, a maybe a photograph of your doctor on the system...And maybe info about postoperative care. Because I mean all these ops have different things and I didn't know I was gonna go to need dietary requirements after the first liver operation...So having that as a portal to kind of find information may be useful*.

[P2]

*I think having someone else who knows, you know, what you've been through would be nice. Yeah. They can give you kind of, like a perspective on what to expect*.

[P4]

*I would prefer to see a video, just a more informal video and then follow up with a verbal conversation just before the operation*.

[P7]

#### PSHR Expectations

Following a brief demonstration of the PSHR, 10 public and 5 private sector postoperative patients with no prior experience of the PSHR (group 2) were interviewed about their expectations of a digital information-sharing tool. Their expectations are summarized in the second part of Table 2, with illustrative quotations provided in Multimedia Appendix 3.

Most participants indicated a preference for accessing the PSHR via a WhatsApp link on their smartphones:

*I think overall on one’s phone is just better, it is more accessible*.

[I2]

*We have emails, but we don’t use it so much*.

[U2]

*Anything that is easy for you is easy for me. But really Whatsapp is easiest*.

[U10]

Participants indicated that they would value features such as curated information, feedback from their surgeon or anesthetist, and the ability to track their recovery progress.

*I don’t want to get the information by doing a google search. I want information that comes from the doctor themselves, so that I know it is correct*.

[I1]

*You know, if I think back to my work again, clients want to be heard… and now in my setting I am not upset about anything, but it may still be nice to be acknowledged, if I fill something in, it would be nice to get a message or a call to confirm that my responses were seen*.

[I3]

*But when you check, keep on checking on your patient, it’s good because if I feel something on me, I have to let you know. Then you'll ask me maybe then to come back at hospital. Then you can check that and sort it out*.

[U3]

*I mean, if they know what your baseline is, what my baseline is, how my life, my, my health is, you know, then they'll know how to proceed. With any procedure for that matter*.

[U5]

Participants also indicated that they would be motivated to use a tool such as the PSHR by knowing that they would contribute data that could help others:

*I would actually do it more for the greater good to contribute to ongoing medical knowledge and learning*.

[I1]

*I think I would still contribute my data if I knew it went for a good cause and if my doctor asked for it*.

[I2]

*If it'll help someone with the same problem that I have, it’s important to share it*.

[I5]

Potential barriers to using the PSHR are high costs of data and low digital confidence:

*When I'm at home, I don't see that airtime. Because it’s a cost of money. It’s very expensive.*

[U3]

*...all the fancy phones, the internet, all that stuff, that’s not for me*.

[U4]

### Phase 2: Define

Insights from the initial interviews were that individuals presenting for surgical procedures have a need for connection with and feedback from their health care providers and a willingness to engage in actions necessary to navigate a challenging phase in their lives. Contrary to the investigators’ expectations, participants who had used the PSHR were not discouraged by the length of the questionnaires, provided they perceived a clear and meaningful purpose to their completion. In addition, patients expressed a need for information related to their surgical procedures, highlighting the importance of incorporating targeted educational content to support informed decision-making throughout the perioperative journey.

### Phase 3: Ideate

**Figure 2.**
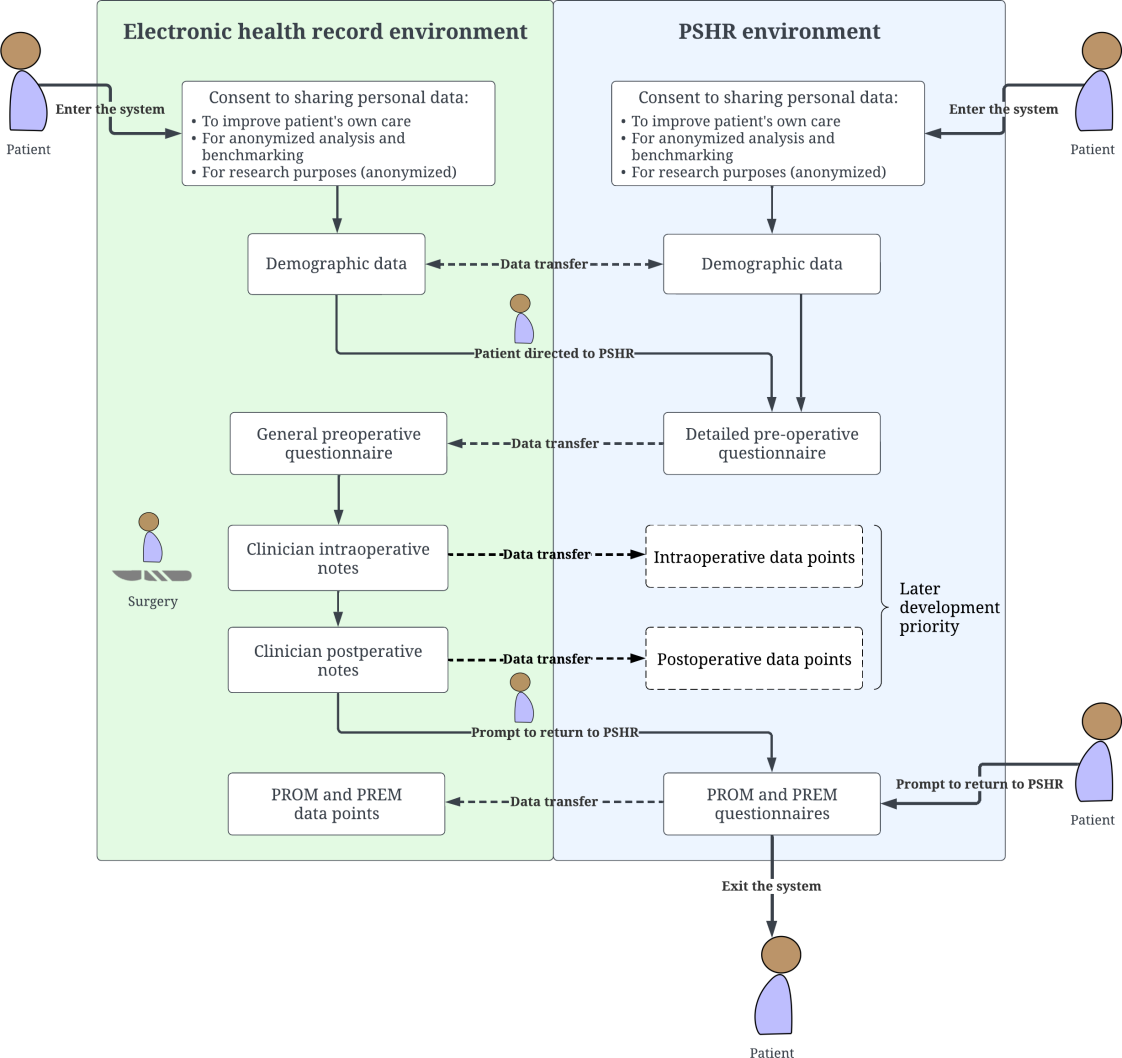
Proposed PSHR interaction and interoperability with electronic health records. PREM: patient-reported experience measure; PROM: patient-reported outcomes measure; PSHR: Perioperative Shared Health Record.

The research team brainstormed suggestions and expectations from patient groups, and how the PSHR can be integrated into usual care processes. While standardized questionnaires remained unchanged, their sequence was reorganized to group similar questions, particularly in the PSHR preoperative questionnaire, where multiple risk assessments and surveys are consolidated into a single comprehensive questionnaire. Feedback messages were developed to provide patients with information tailored to their questionnaire responses. Various approaches were explored to support patient-clinician communication through the PSHR. The research team also considered the potential interaction of the PSHR with electronic health records (Figure 2).

### Phase 4: Prototype

A series of low-fidelity wireframes were created based on the potential solutions obtained during phase 3. These wireframes were refined into a low-fidelity paper prototype of the PSHR.

### Phase 5: User Test

**Figure 3.**
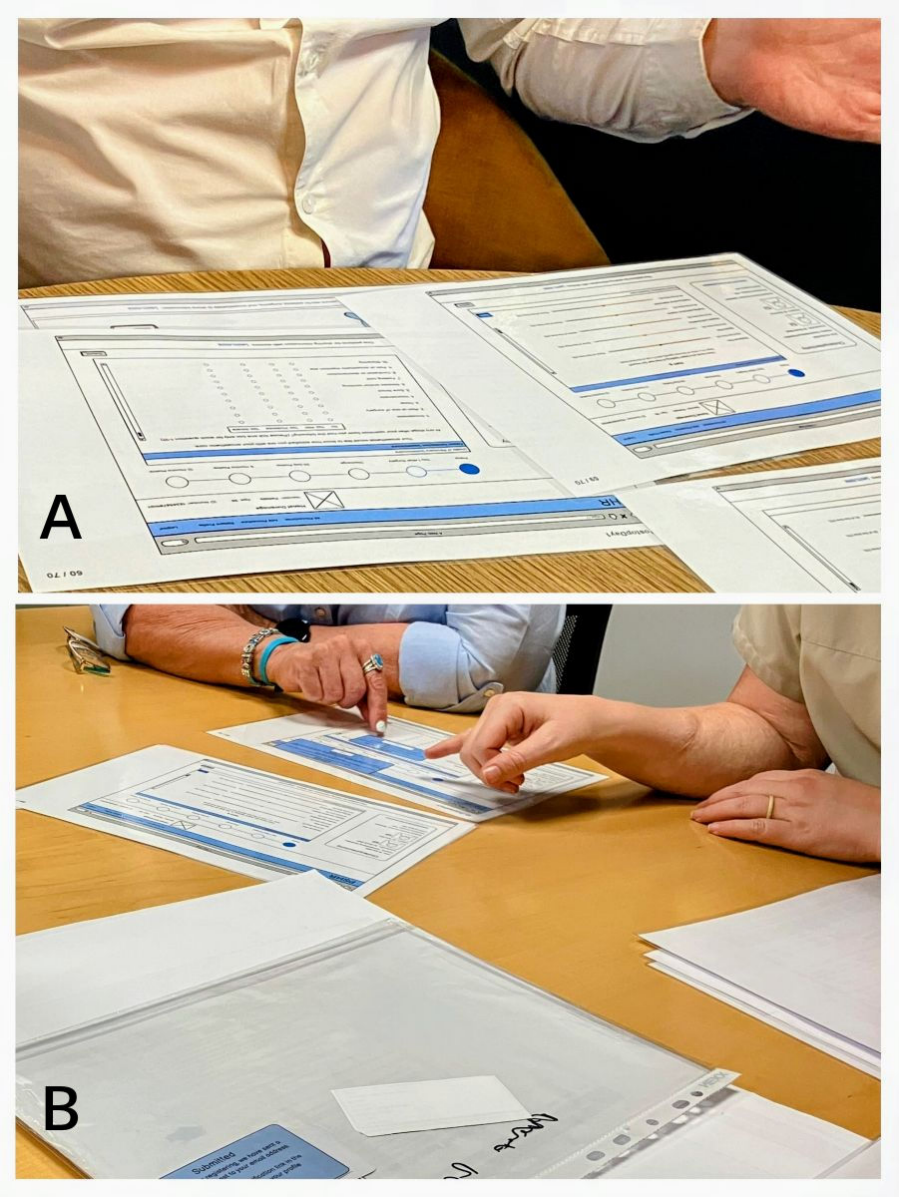
Paper prototype testing in action. (A) A participant discussing task 2 (completing the preoperative questionnaire). (B) A participant responding to a pop-up notification during task 4 (completing postoperative questionnaires).

Five individuals consented to participate in prototype testing. Three participants had previously used the PSHR (“expert users”), and 2 participants had no prior experience of the PSHR (“novice users”). Five users have been reported as sufficient for undertaking user testing [58,59]. Demographics are summarized in Table 3. All participants reported having their own mobile phone and usually accessing the internet and their email on their mobile phone and not on a computer. Each testing session took approximately 60 minutes to complete, with the most time spent on the second task. Figure 3 shows paper prototype testing in action; Figure 3A shows a participant discussing task 2 (completing the preoperative questionnaire), and Figure 3B shows a participant responding to a pop-up notification during task 4 (completing postoperative questionnaires).

**Table 3.** Demographic data for participants taking part in phase 5: prototype testing.

ID^a^	Age (years)	Sex	Language	Race	Prior PSHR^b^ experience	Education	Surgery
T1	42	Female	English	White	Yes	After school qualification	Major abdominal
T2	63	Female	Afrikaans	White	Yes	After school qualification	Major abdominal
T3	46	Male	Afrikaans	White	Yes	After school qualification	N/A^c^—assisted family member
T4	21	Male	Northern Sotho	Black	No	After school qualification	Head and neck
T5	32	Male	Setswana	Black	No	Secondary school completed	Head and neck

a User ID explanation: “T” denotes participant responses in the user testing phase.

b PSHR: Perioperative Shared Health Record.

c N/A: not applicable.

For the first task (registering and consenting), trust was an important factor for 2 of the participants. Prior notification by their doctor to expect a registration email would help improve trust when receiving a link to an unknown website:

*Yeah, knowing that this is safe, yes, because I spoke to you and I know that you will give me something like this. I think a personal call, direct, to say I'm sending you something now. I trust it more, because I know that I'm protected*.

[T4]

Two of the participants read the consent form in detail, 2 participants scrolled through with minimal reading, and 1 participant indicated that he would abort the process when confronted with a lengthy consent process:

*Unless if I’m buying a car, I don’t read the details*.

[T1]

*As soon as I get this information, when I get to this one, I will say, yoh aha, this is too much. Pause, pause, pause*.

[T4]

The 2 participants who read the consent form doubted that most users would engage deeply with the consent process. One participant suggested that using illustrations or icons could clarify abstract concepts. During the second task (preoperative questionnaire), various data input methods were tested, including radio buttons, colored numerical sliders, and free text blocks. Users seemed to appreciate color coding to interpret the numerical sliders. One participant suggested modifications to the order of questions.

Participants were able to navigate to the information portal (task 3); all participants found general information links useful but preferred procedure-specific content, with 3 favoring video links over text, and 1 mentioning that they would refer to written information only if the desired content was not available in the video links.

By the fourth task, participants were familiar with the layout of the home page and the questionnaires. Their understanding of the timeline had improved, but several suggestions were made to enhance its visual clarity. Feedback messages following questionnaire completion elicited mixed responses; some participants expressed concern that alerts about poor recovery outcomes could cause anxiety:

*It makes me feel worried...I will go back to what I completed [to check] that I completed it correctly. Okay. Because there might be something that I said that might alarm the system.*

[T5]

All participants indicated that they would value automatic feedback from their surgeon or anesthetist if they recorded poor scores on their postoperative questionnaires, with expectations for response times ranging from 30 to 60 minutes to up to 48 hours. Participants also noted that a lack of clinician feedback would reduce their motivation to continue using the platform. The potential for the PSHR to enhance patient-doctor relationships is illustrated in this quote:

*So you still need to get to the buy-in from this. Where’s my buy-in coming from? It’s coming from the aftercare service, from the doctor, building that relationship. Because [of feedback through the PSHR] I've got a relationship again with the doctor, the surgeon or the rooms. I would be their patient for life! I think that’s for me, that’s how you get the buy-in to carry on the rest of the process.*

[T2]

**Figure 4.**
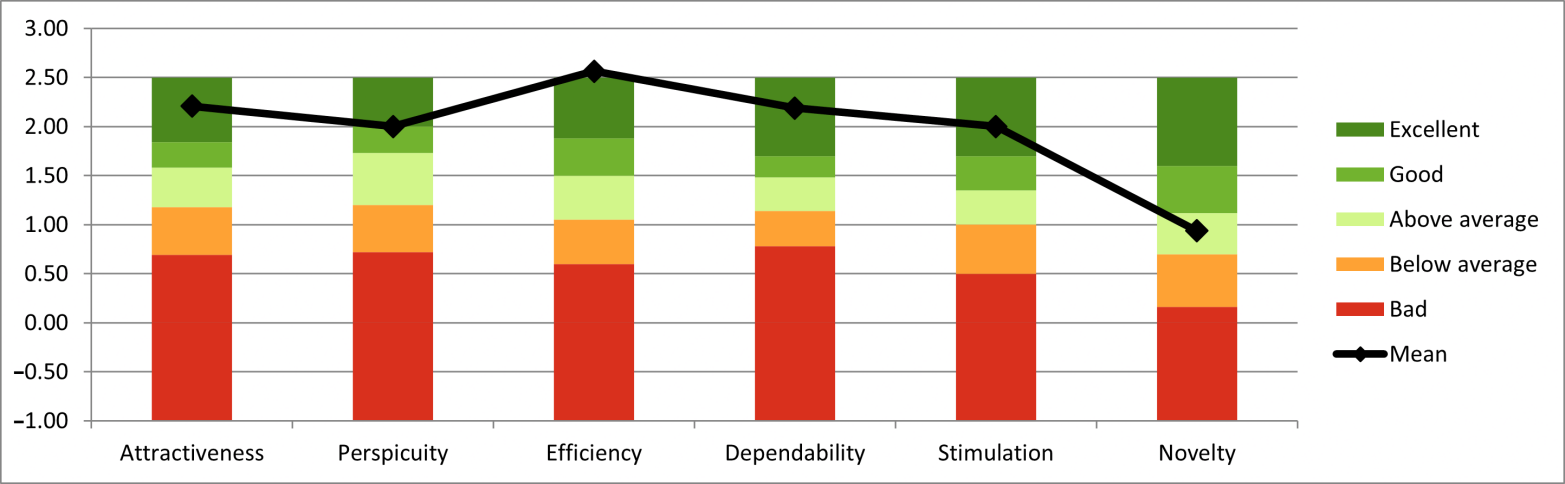
Comparing the Perioperative Shared Health Record (PSHR) paper prototype User Experience Questionnaire scores to benchmark data. The measured scale means from our study are compared in relation to existing values from a benchmark dataset, which allows conclusions about the relative quality of the PSHR paper prototypes according to user testing compared with other products.

Four participants (3 experts and 1 novice) completed the SUS and UEQ questionnaires related to the paper prototype testing. The overall mean SUS score was 91.3 (SD 5.7), which indicates very good usability. The mean SUS scores per usability aspect were learnability 91.3 (SD 10.2), efficiency 93.4 (SD 6.9), and satisfaction 89.1 (SD 6.8). The UEQ mean scores for the attractiveness scale are 2.21 (SD 0.6), perspicuity 2.0 (SD 0.9), efficiency 2.6 (SD 0.4), dependability 2.2 (SD 0.6), stimulation 2.0 (SD 0.8), and novelty 0.94 (SD 1.4). The UEQ scores ranged from good to excellent, except for novelty, which scored above average (Figure 4).

## Discussion

### Principal Results

This study applied a human-centered design approach to evaluate and improve patient user experience of a digital health tool developed to capture perioperative patient-reported outcomes. While identifying key usability challenges, it also showed how digital health tools such as the PSHR can help enhance connection between patients and clinicians through information sharing and timely feedback. The findings contribute new evidence from an LMIC setting, where practical integration of PROs into perioperative care is limited. By drawing on patient experiences as a resource for design, the study demonstrates how patient involvement can inform iterative improvements to digital health tools and strengthen person-centered perioperative care [7,21,22,60,61].

The findings of our study align with established user experience principles in digital health design, emphasizing the importance of empathy, communication, accessibility, regulatory compliance, and data privacy and security [21].

Mapping the patient journey revealed the emotional strain of the perioperative period and the value of designing digital health tools that provide empathetic support during this vulnerable time [21,60]. This aligns with previous research highlighting that emotional engagement and good information provision are central to person-centered perioperative care and patient satisfaction [1,62,63]. Experiences reported in HIC show that access to targeted digital health tools can improve patient well-being and empowerment as well as improve postoperative outcomes [18,61,63,64]. Providing patients with a digital resource that offers clear, accessible information may therefore strengthen patient engagement by improving understanding of recovery and fostering a sense of partnership in care [1,18,65].

A key finding was the importance of communication and feedback from clinicians to create trust and to maintain motivation to continue using the system. This aligns with studies on implementation of electronic health records and patient perspectives on digital health tools [61,66]. It was interesting to note that patients were not deterred by lengthy questionnaires if they perceived them as purposeful. Participants valued knowing that their submitted data would inform their care, similar to evidence that perceived purpose and clinician responsiveness may increase adherence to digital platforms [61,64,67]. However, clinicians may not always see the value of using digital health tools to strengthen relationships with patients, especially if these tools are perceived as adding to their workload [28,66,68]. It is important to note that for patients who used the PSHR, a lack of clinician feedback following the completion of the postoperative questionnaires reduced their motivation to continue engaging with the tool. Therefore, it is important that patient needs are balanced with clinical feasibility. One potential solution would be to use automated alerts from PROM data that notify clinicians when patient responses are below a predefined threshold, prompting timely feedback to those patients who need them. This could enhance the perceived usefulness and reliability of the PSHR and strengthen the patient-clinician relationship, without overburdening the clinician [1].

Using the PSHR to track and benchmark recovery progress can offer reassurance or prompt patients to seek help when needed. Such features can promote self-management by helping patients to understand their recovery and to feel more in control of their health [61,64,67]. From our user testing, it emerged that when automated feedback messages to patients flag potential concerns, these messages should balance the communication of information with reassurance and clear guidance on next steps.

Despite the benefits of using a digital tool such as the PSHR, barriers such as limited digital literacy, the high cost of data, and inconsistent access to internet connectivity are significant obstacles to digital health implementation in South Africa [33,39] Patient preference for accessing the PSHR via WhatsApp links highlights the importance of incorporating widely used, low-barrier communication channels in LMIC settings. This aligns with priorities outlined in the South African Digital Health Strategy and the WHO Global Strategy on Digital Health, which highlights the need for equitable access, user-centered design, and interoperability of digital health tools [20,69].

Regulatory constraints, particularly around the consent process, present a design challenge for the PSHR. While simplifying consent forms with icons and condensed text may improve accessibility, maintaining careful attention to detail and to legal and ethical standards is important to ensure the integrity of the consent process. Providing reliable and up-to-date medical information is resource-intensive, whether creating original content or vetting existing material. One potential solution is to involve patients in content development and curation, fostering a collaborative platform. However, ensuring the accuracy of medical content would still require professional oversight and quality control.

An additional consideration in the design of the PSHR is safeguarding data privacy and security, especially as it collects personal and health information subject to the South African Protection of Personal Information Act, comparable with the Health Insurance Portability and Accountability Act in the United States and the General Data Protection Regulation in Europe. In the South African private health care sector, access to patient data can be restricted to the patient and their designated surgeon and anesthetist, which enhances data security. However, in the public health care sector, where care is provided by teams rather than individuals, maintaining data privacy may be more difficult. Concerns about cybersecurity and a lack of trust in an unknown system were seen by some patients as barriers to engaging with the PSHR.

### Strengths and Limitations

A key strength of this study lies in its adherence to design thinking and human-centered design principles [21,22]. The use of Karagianni’s Optimized Honeycomb model provided a structured lens for analyzing user experience and expectations, capturing functional cognitive and emotional factors influencing user experience [47]. Involving individual patients in the design process enabled the research team to draw on their lived experience as a form of expertise. By prioritizing the needs of actual patients rather than relying on personas, we aim to advance our mission of developing an intuitive, efficient, user-friendly, and person-centered tool. Furthermore, the inclusion of a diverse sample of patients from both the public and private health care sectors in South Africa enhances the study’s relevance, particularly as the country progresses toward universal health coverage. Including in the research team a clinician focused on patient-reported outcomes and a hospital patient representative kept the group focused on a patient-centered approach.

The main limitation of this project is the inclusion of a relatively small patient sample across the various phases, limiting the generalizability of findings. However, from a user experience research perspective, idea saturation in initial interviews suggests sufficient theme coverage, and prototype testing revealed consistent usability issues, aligning with Nielsen and Landauer’s 5-user rule [58,59]. The original study protocol aimed to include patients from Sweden to allow comparisons with a high-resource setting, but logistical and resource constraints prevented this. Future research will focus on expanding data collection and user testing, with a comparative analysis between HICs with well-established digital health platforms and LMICs where digital health systems are still being developed. An additional limitation was that user testing was conducted only with low-fidelity paper prototypes. However, this is a recognized approach within design thinking methodology, enabling iterative development without significant cost investment during the early stages when design elements remain subject to change [23,25,70].

Considering that one of the researchers has a professional interest in PROs and had established rapport with several participants, this may have introduced a subtle positive bias in how participants perceived and articulated the value of the PSHR. Furthermore, as the majority of patients had undergone intermediate or major surgical procedures, their emphasis on the need for information and emotional support may not be generalizable to patients presenting for minor operations.

Another limitation is that the study does not capture the user experience of health care providers. The original project plan included workshops with surgeons and anesthetists; however, time constraints necessitated postponing these activities. Ongoing work by the South African authors includes intentional network weaving to promote data-driven surgery which will include engagement with perioperative clinicians.

Although initially unfamiliar to the anesthesiologist investigators, the qualitative and user experience methodologies provided valuable learning. This collaboration highlighted the importance of such approaches in helping clinicians understand patient needs and to develop intuitive digital health tools.

### Future Research

Findings from this study will inform further design iterations of the PSHR, both to optimize its use in individual patient care and to generate future research outputs. Next steps include testing a high-fidelity prototype and evaluating the final product in real care settings, with particular attention to patient experiences over time: for example, how patients respond to repeated questionnaires that may appear similar at different intervals. Data from the PSHR will in time become a resource for organizational development and quality improvement. Ongoing development will require the active involvement of both clinicians and patients to ensure that the tool remains relevant, feasible, and responsive to real-world clinical processes and workflows.

### Conclusions

This study is one of the first to apply human-centered design principles to a perioperative digital health tool in an LMIC setting, addressing usability challenges and patient engagement. Key user experience factors influencing patient engagement included communication, feedback, and access to information throughout the surgical journey. Digital health tools such as the PSHR can strengthen communication and support person-centered perioperative care by integrating PROs into clinical workflows and care processes. As health care systems worldwide move toward digital integration, our findings provide valuable insights into the factors to consider when digital health tools are introduced in diverse health care contexts. Future research should focus on integrating digital health tools into clinical workflows and assessing their impact on person-centered outcomes and care delivery, with particular emphasis on involving all relevant stakeholders, both clinicians and patients, to ensure that the tools are contextually appropriate and aligned with real-world processes and workflow needs.

## Supplementary material

10.2196/79349 Multimedia Appendix 1 Patient semistructured interviews guide.

10.2196/79349 Multimedia Appendix 2 Key tasks during paper prototype testing.

10.2196/79349 Multimedia Appendix 3 Detailed thematic analysis.

## Abbreviations

HIC: high-income country
LMIC: low- and middle-income country
PREM: patient-reported experience measure
PRO: patient-reported outcome
PROM: patient-reported outcome measure
PSHR: Perioperative Shared Health Record
REDCap: Research Electronic Data Capture
SACSOS: South African Collaborative Surgical Outcomes Study
SUS: System Usability Scale
UEQ: User Experience Questionnaire
