# Supplementary material for: Enhancing the User Experience of a Perioperative Digital Health Tool for Information Exchange Using a Human-Centered Design Thinking Approach: Qualitative Observational Study
Source: JMIR Perioper Med. 2026 Jan 12;9:e79349. doi: 10.2196/79349 (PMC12795411; doi:10.2196/79349)
Supplement: Multimedia Appendix 1 [file periop-v9-e79349-s001.docx]

## Multimedia Appendix 1. Interview questions

These questions may be used during one-on-one interviews to focus and guide discussions.

| **Demographic information** | | |
| --- | --- | --- |
|  | Age, sex | |
|  | Country, City, Home Language | |
|  | Level of education / Literacy level | |
|  | Previous exposure to the PSHR? | |
| **Perioperative experience** | | |
|  | Tell us about your experience as a patient who needed/needs surgery. | |
|  | Imagine you had to go for your surgery again, what information would you like to have had before the surgery? | |
|  | What was good about your experience? | |
|  | What was not good about your experience? | |
| **General Digital Literacy** | | |
|  | What device do you normally use to access the internet? | |
|  | Do you have internet at home / work / school / when moving around? | |
|  | Have you used any digital health applications or tools before? | |
|  |  | If yes, what motivated you to use the application or tool? |
| **PSHR specific questions (if prior experience with the tool)** | | |
|  | What motivated you to use the PSHR? | |
|  | What did you like about using the PSHR? | |
|  | What did you not like about using the PSHR? | |
|  | When you completed a questionnaire, what did you expect would happen next? | |
|  | Would you like it if the PSHR gave you a message to say how your questionnaire input compares to other patients who had similar surgeries? | |
|  | Would you like it if the PSHR had links to information regarding what to expect for your type of surgery? Would you prefer this information to be a video link or a downloadable text document? | |
|  | Would you like it if the PSHR sent a message to your surgeon or anaesthesiologist when you are worried about some of the responses you gave? | |
|  | Is there anything else that you can suggest to improve the PSHR? | |
| **PSHR specific questions (if NO prior experience with the tool, after short demonstration of the tool)** | | |
|  | Do you think this is a useful tool for you as a patient? | |
|  | What would motivate you to use a platform like this? | |
|  | What is missing from the platform? What would you like to see in a platform like this? | |
|  | Would you like to see video content or images or text based information relating to your condition / procedure? | |
|  | How would you prefer to access the platform? Via the internet / web-based application, or via an app on your phone? | |
