# Supplementary material for: Enhancing the User Experience of a Perioperative Digital Health Tool for Information Exchange Using a Human-Centered Design Thinking Approach: Qualitative Observational Study
Source: JMIR Perioper Med. 2026 Jan 12;9:e79349. doi: 10.2196/79349 (PMC12795411; doi:10.2196/79349)
Supplement: Multimedia Appendix 2 [file periop-v9-e79349-s002.docx]

**Multimedia Appendix 2.** Key tasks.

| **Instruction** | **To Do** | **Desired Outcome** |
| --- | --- | --- |
| - You are a patient who will be going for a surgical procedure. - Your surgeon has asked that you complete a questionnaire about your general health before you come for the surgery. - They have sent you a link via email to the website where you will complete the questionnaire. | 1. Follow email link 2. Register on PSHR 3. Verify email 4. Consent 5. Get to the dashboard where the procedure is loaded. | 1. Register 2. Get to dashboard |
| - You now need to complete the preoperative questionnaire.   - Your weight is 80kg, your height is 175cm   - You have hypertension   - You take amlodipine 5mg for your hypertension   - You are allergic to aspirin   - You don’t smoke or drink   - You are otherwise healthy   - You have not had a surgical procedure before | 1. Complete the different sections of the questionnaire. | 1. Complete all the sections of the questionnaire 2. Go back to the dashboard |
| - You want to get some more information on your procedure. | 1. Navigate to the Information Portal | 1. Get to the navigation portal 2. Get back to dashboard 3. Can log out now |
| - You have had your surgery, it is the day after your procedure. The anaesthetist asks you to please complete your Day 1 questionnaires on the PSHR. | 1. Complete the quality of recovery questionnaire 2. Complete the patient satisfaction questionnaire | 1. Log in 2. Go to dashboard 3. Navigate to Day 1 after surgery 4. Complete quality of recovery and patient satisfaction questionnaires 5. Get back to dashboard |
