# Supplementary material for: Enhancing the User Experience of a Perioperative Digital Health Tool for Information Exchange Using a Human-Centered Design Thinking Approach: Qualitative Observational Study
Source: JMIR Perioper Med. 2026 Jan 12;9:e79349. doi: 10.2196/79349 (PMC12795411; doi:10.2196/79349)
Supplement: Multimedia Appendix 3 [file periop-v9-e79349-s003.docx]

**Multimedia Appendix 3**

**Focus group 1: PSHR Exposed patients (private healthcare sector).**

*Participants identified as “P” and a number according to the sequence in which they were interviewed.*

| **Main Theme** | **Subtheme** | **Code** | **Code Count** | **Case Count** | **Quote** |
| --- | --- | --- | --- | --- | --- |
| Patient journey  *How did the patient experience the time before and after their procedure?* | Information seeking behaviours  *What preparation did the patient undertake before their procedure?* | Doing own research | 1 | 1 | “I was fine doing my own searching really. Um, but then I think it's, it's the researcher in me as well.” P1 |
|  |  | Information from family members | 1 | 1 | “So I had my sister-in-law, who is a general practitioner, check for the results because I wasn't gonna wait for him. And then she was the one that was, I think, it was traumatic for her as well. Because you don't wanna have that conversation with family.” P2 |
|  |  | Information from other patients | 1 | 1 | “And I was also following, uh, people that will talk about their experience, you know.” P1 |
|  |  | Avoidance | 3 | 3 | “I don't really want to Google stuff because you always, there's always stuff. Too much information. I googled my, my, um, situation December last year and I realized I need to get to a doctor because, um, you know, before you scare yourself, this is not normal. I can't wait any longer.” P3  “I think that would've scared me off a little bit more if I knew truly what was to come.” P4  “No, for me the way that [the anaesthetist] and [the surgeon] did it was ideal. I wouldn’t have watched a video or read anything else at that stage… I would actually have asked if it is really necessary for me to do additional reading, what is the benefit?” P5 |
|  | Emotional response  *What was the patient’s emotional response before their procedure?* | Denial | 3 | 3 | “[My wife] and I were visiting [our daughter], and I started to have stomach pain, and I didn’t want to say anything, but it got worse over a few days, and then I started to vomit, and then [my daughter] said we have to go to hospital.” P5 |
|  |  | Emotional turmoil | 7 | 4 | “But now it all became too much. It just felt like you take one step forward and like five steps back and one step forward and five step back.” P1 |
|  |  | Frustration at process | 5 | 4 | “I was kind of upset because I was just like lying here thinking on like, oh, when is this going to, you know, eventually get better? It felt like such a long time.” P4 |
|  |  | Helplessness | 1 | 1 | “It felt like I was in constant pain and I also felt very helpless.” P1 |
|  |  | Hopeful | 4 | 2 | “And she said to me, no, not at all! But that answer that one sentence, and with such conviction, uh, brought back my, um, my hope.” P3 |
|  |  | Isolation | 2 | 2 | “It was very, because nobody can come in with you and then you're there alone and then they don't communicate well doctors all the time, some of them.” P2 |
|  |  | Relief | 1 | 1 | “But, um, it did feel, I felt better when we did find out. When you find out, yeah. There's a bit of a relief, you know, there is a problem we can do something about it.” P1 |
|  |  | Worried | 1 | 1 | “My obviously concern was his age. My dad is now 72, and also about the risk of an operation.” P7 |
|  | Postoperative difficulties  *Difficulties that the patient had after their procedures.* | Confusion | 3 | 2 | “And if you have medication on board and you, um, you thinking they're talking about you.” P3 |
|  |  | ICU difficult place to be | 2 | 2 | “I think I, I don't remember when I first woke up, but I do remember, um, the other days it was kind of, it was kind of hard.” P4 |
|  |  | Pain and physical discomfort | 5 | 2 | “But I remember I woke up in so much pain, you know, so much pain.” P1  “I think the first two weeks were the hardest really. And the vomiting was much worse at home. Yeah. Uh, the pain also from eating was really terrible.” P4 |
|  | Interaction with healthcare providers  *Patient thoughts on the interaction with their health care team.* | Relationship with patient | 7 | 3 | “But [the surgeon] had his calmness about him, um, and that made me calm. He was not anxious or, you know, he didn't say too much what he said was enough.” P3 |
|  |  | In person communication | 10 | 6 | “It made such a difference that [the anaesthetist] were there and [she] could explain to us what was going to happen, it made us feel a lot more secure and calm.” P6 |
|  | What made the journey easier?  *Things that made it easier for patient to cope during the perioperative period.* | Being informed | 1 | 1 | “So being informed Yeah. Makes you feel more reassured.” P3 |
|  |  | Family support | 1 | 1 | “If the people around you, if you've got this support, my husband was so supportive.” P3 |
|  |  | No delay to treatment | 1 | 1 | “I think everything moved quite fast once we got to [hospital] because they were able to tell me exactly what it is. “ P4 |
|  |  | Early mobilisation after surgery | 1 | 1 | “I stood up on the side of my bed within 24 hours, the physio came, he was talking to me, they distract you like with a carrot. And the next thing I know he pulled me up standing. I was like, how am I doing this? Which I think helps a lot.” P2 |
|  |  | Quick Recovery | 1 | 1 | “And then, um, from there on, it was two days and I was out of hospital. I think I was in hospital only for a week.” P3 |
|  | Advice to other patients  *Things the patient would have liked to know before going for surgery.* | Consider the emotional impact | 2 | 2 | “It's the emotional side of things that takes quite a toll. But not only on [the patient], but on my [the family] too. So I think the emotional strain on both was tough.” P7 |
|  |  | Physical preparation | 2 | 1 | “Do lung exercises! I actually saw the physio six weeks before I went in for the last surgery and she gave me that device. She calls it weights for your lungs.” P2  “You know, the moment you can go to the bathroom, get to the bathroom and you, you just feel like a person again.” P2 |
| PSHR Experience  *How did the patient experience using the PSHR?* | Use  *Using the product or application.* | Accessible  *Access to a device the patient will use the PSHR on*. | 10 | 6 | “I do prefer that it was easy to use on my phone. So if it can be improved, it must still just be improved. Mainly for, for like a smartphone.” P1  “It was a bit difficult. Different. If you wear glasses and you don't have glasses on, and you're on morphine. But it wasn't, it wasn't impossible.” P2  “No, I’m not so comfortable with my phone. The internet on there is not something that I usually use.” P5  “…all the fancy phones, the internet, all that stuff, that's not for me.” U4 |
|  |  | Findable  *How easy it is for patients to navigate to the platform.* | 15 | 5 | “No emails get lost. Yeah, absolutely. I have in my work email, I have over 700 unread ones. My Gmail is probably 9,000. Yeah. I still, I still pay attention to WhatsApp.” P2  “Yes, because on personal email you're not visiting that often, so it gets lost with all the other stuff. So I believe your preferred communication is WhatsApp” P7 |
|  |  | Usable  *Ability to use the application and how information is perceived.* | 24 | 6 | “it just went on and on and on. But I think at the time as well, 'cause um, we met, just before I was about to give birth. I think we met the year I was about to give birth. Um, I, I think in my head it was just all part of, just part of the process preparation and the process you had to, you know, and making sure that everything is fine” P1  “If I didn’t get feedback, I wouldn't have filled in anymore. I would've done the first one and left it that.” P2  “There's some, um, uh, of the wordings and stuff that I really didn't understand.” P3  “It was easy to answer. It doesn't take too long.” P4  “I think the only complaint if I need to complain about improvements will be the size of the font perhaps. And on my phone I couldn’t see everything, so I had to scroll and drag the screen to see the full question. So I think the font size perhaps needs to have some adjustment.” P7  “Immediate moment? Um, the one month in the three months? Yeah. I think, uh, the sixth month and the 12th month, just not that much.” P1 |
|  | Feel  *Emotional response users have when using the application.* | Desirable  *Features that would fulfil a need and make the user want to use the product.* | 2 | 2 | “You have more thoughts and it's more logical to fill in online when you are sitting at home. It's not like the day of the surgery. So you're more focused.” P2 |
|  |  | Credible | 0 | 0 |  |
|  | Think  *Users’ cognitive engagement with an application.* | Useful  *Does the application fulfil a user need?* | 28 | 7 | **Feedback**: “[The anaesthetist] had read what was going on. She came and she asked what was going on, and I explained that and she worked around it and talked to the staff.” P1  **Communication**: “That was the other thing that was very important with the survey I managed to put in, please keep me elevated after the op. Yes. because my lungs collapsed after the first operation. And I also believe that made a huge difference in how I felt after the surgery.”P2  **Benchmarking**: “all of this is quite relevant because it lets you think about your own wellbeing and progress.” P6  **Access to HCP:** “And that might trigger me calling the doctor and say, actually, I've got a pain. And maybe that will lead to a consultation if needed.” P7 |
|  |  | Valuable  *Whether the platform as a whole delivers value to the user.* | 7 | 4 | **Improved Care**: “I suppose that was proved to me, because in many cases I would've gone through that, and cried myself to sleep. And there was no way of my doctor ever knowing what I went through. But I suppose with, with that, then [the doctor] was able to quickly know and, and come back and improve the, and improve my care, you know?”P1  **Engagement**: “You feel that you, you were kind of more involved in the planning of your care” P2  **Altruism**: “…if my information can help somebody else, to get through a very difficult situation … then I feel, you know, it's worth it.” P3 |
|  | Enhancements  *What would the user suggest to improve the PSHR?* | Ability to enter free text | 2 | 2 | “having the ability to have free form open text. Yeah. If somebody wants to say something more.” P2 |
|  |  | Ability to contact other patients | 3 | 3 | “I think having someone else who knows, you know, what you've been through would be nice. Yeah. They can give you kind of a like, perspective on what to expect.” P4 |
|  |  | Information about the healthcare practitioner | 3 | 2 | “I suppose especially for, for large operations, it might help people to know who their anaesthetist is and have like a name and a, a maybe a photograph of your doctor on the system. “ P2 |
|  |  | Information on postoperative diet | 1 | 1 | “And maybe info about postoperative care. I know it's not really anaesthetist thing but what, like, um, what would eat, because I mean all these ops have different things and I didn't know I was gonna go to need dietary requirements after the first liver operation.” P2 |
|  |  | Information on what to expect | 1 | 1 | “…having kind of something that said, it's gonna be awful, but there's a time limit to it with something like that?” P4 |
|  |  | Information Portal | 6 | 3 | “I read a lot. If you tell me there's something wrong with me, I'm going to read like 60 million things about everything, you know.” P1  “So having that as a portal to kind of find information may be useful.” P2  “I would prefer to see a video, just a more informal video and then follow up with a verbal conversation just before the operation.” P7 |
|  | Barriers | Emotional overwhelm | 2 | 1 | “Mmm, I must say, there are so many links and things that the medical aid sent beforehand, that I think if there was any additional information to look at or links to click on it would actually have been a bit too much.” P6 |

**Focus group 2: PSHR nonexposed (private healthcare sector).**

*Participants identified as “I” and a number according to the sequence in which they were interviewed.*

| **Main Theme** | **Subtheme** | **Code** | **Code Count** | **Case Count** | **Quote** |
| --- | --- | --- | --- | --- | --- |
| Patient journey  *How did the patient experience the time before and after their procedure?* | Information seeking behaviours  *What preparation did the patient undertake before their procedure?* | Information from health care provider | 4 | 3 | “I listened to what the doctor said, and I looked at the list of things they said I had to bring along to the hospital, but I didn’t really want to go looking for more information, cancer taught me that I think.” I3 |
|  |  | Information from coworker | 2 | 1 | “But my team leader said, but you are supposed to be at hospital when I read your story. I said, I'm afraid to go there because they want to take a piece of the bone. He said to me, he advised me, no, it's better and see for you to go there whie this problem is still early because we don't know what is the cause of the problem. Stop running from healing.” I5 |
|  |  | Information from other patients | 4 | 3 | “I must say the information from other patients helped a lot, knowing what someone else went through, their experiences, how they felt, what the cost implications were, how they paid it, all of that helped a lot.” I2 |
|  |  | Avoidance | 1 | 1 | “because you know when you google things you don’t always get the right information. And it can be very scary.” I3 |
|  | Emotional response  *What was the patient’s emotional response before their procedure?* | Anxiety | 1 | 1 | “Well, you know, I saw during my cancer journey, that there were some patients who worked themselves up so much, and they got so anxious by looking things up on the internet.” I3 |
|  |  | Fear | 1 | 1 | “I started like shaking and getting worried. Yes. Like now it’s getting worse. And like I took it easy, like okay, fine. I went back to work instead of going to the doctor.” I5 |
|  | Postoperative difficulties  *Difficulties that the patient had after their procedures.* | Complications | 1 | 1 | “…And it affects my personal life, my work.” I5 |
|  |  | Diet restrictions | 1 | 1 | “I must say the hardest thing for me was having to go onto pureed food, not being able to eat, and even now afterwards it will be at least another month before I can eat solid food again. It’s quite a shock to the system, it’s a drastic lifestyle change that you have to make before you come for surgery.” I2 |
|  | Interaction with healthcare providers  *Patient thoughts on the interaction with their health care team.* | Physiotherapist important role | 1 | 1 | “And then the physio explained nicely about how I should come for follow-ups and the exercises that we should do.” I1 |
|  |  | Recommendation and reputation | 1 | 1 | “Yes, I heard about him, and his child was in the school where I teach, and I had heard that he had a good reputation. And when I had the ultrasound, the ultrasonographer recommended him as well when I asked her.” I1 |
|  |  | Time spent in person valued | 2 | 2 | “It was the first time that an anaesthetist really took the time to explain things to me nicely. Anaesthetists usually just see one very quickly right before the operation. And this anaesthetists by contrast came well before, and asked me what my concerns were, what was I worried about, and took the time to explain to me what he was going to do.” I1 |
|  |  | Conflict with HPC | 2 | 1 | “Whereby the doctor like now starting to be rude. It's like I'm not serious about healing and then he can leave me anytime. He’s not a good doctor, according to my thinking and the way he treated me.” I5 |
|  | What made the journey easier?  *Things that made it easier for patient to cope during the perioperative period.* | 24hr access to number for surgeon | 1 | 1 | “The interesting thing about this surgeon’s practice, that I have not come across before, is that he gives you a 24hr whatsapp nr that you can use any time of the day if you have problems or questions. There is always someone that responds – that is not something that everyone would do.” I4 |
|  |  | Having a bath | 1 | 1 | “When they gave me a bath after the procedure! I know it sounds terrible, but after the operation, I couldn’t move, I couldn’t wipe my face, and there was this goo on my face, and it was 24hrs later when I could finally have a bath, and then I just felt better.” I3 |
|  |  | Contact with family | 1 | 1 | “And then also, when I had my phone, I could get in touch with my two kids, which also made me feel better.” I3 |
|  |  | Walking | 1 | 1 | “And then when I could start walking on my own.” I3 |
|  | Advice to other patients  *Things the patient would have liked to know before going for surgery.* | Due diligence | 1 | 1 | “And secondly, and the doctors must be sure about what they are going to do.  Because like as I was saying, in my case, I shouldn't have been here if those doctors there have looked at things very carefully.” I5 |
|  |  | Patience | 1 | 1 | “As a patient, I will say first thing first you need to be patient. You need like, healing is a mercy. It won't just happen overnight.” I5 |
|  |  | Listen to doctor | 2 | 2 | “I would tell them that it is very important to listen to what the doctor tells you. To stick to the rules... And I would tell them that they shouldn’t be scared to go through with it.” I1 |
|  | Concerns | Missing work | 3 | 2 | “My main concern was how long I would have to be away from work, I couldn’t be away from work for 6 weeks at a time.” I3 |
|  |  | Cost | 5 | 4 | “Everyday I discover something else that the [medical scheme] wont pay for." I4 |
| PSHR Expectations  *What expectations would the patient have for a digital health tool like the PSHR?* | Use  *Using the product or application.* | Accessible  *Access to a device the patient will use the PSHR on*. | 6 | 4 | “But what I will say is that I only got my phone back on the 3rd day after my operation, which was yesterday, before that I didn’t have access to it, because I was in the multi ICU.” I3  “I think overall on one’s phone is just better, it is more accessible.” I2 |
|  |  | Findable | 0 | 0 |  |
|  |  | Usable | 0 | 0 |  |
|  | Feel  *Emotional response users have when using the application.* | Desirable | 0 | 0 |  |
|  |  | Credible  *Do users feel that the platform is safe to use and if the information in the platform is trustworthy?* | 1 | 1 | “…especially in the ICU, one is so dependent on the nurse who is looking after you, I would be worried if I say something that isn’t good, that it would come back to the nurse looking after me, and that it may affect my care in the end. That there may be retaliation.” I2 |
|  | Think  *Users’ cognitive engagement with an application.* | Useful  *What needs should the platform address?* | 10 | 4 | **Curated list of information**: “I don’t want to get the information by doing a google search. I want information that comes from the doctor themselves, so that I know it is correct.” I1  **Efficiency**: “What I also liked, is that the day before, I got a link to complete the anaesthetic forms online, and it was very quick and professional and efficient. So I knew my information is already shared and a connection was established” I1  **Feedback**: “You know, if I think back to my work again, clients want to be heard… and now in my setting I am not upset about anything, but it may still be nice to be acknowledged, if I fill something in, it would be nice to get a message or a call to confirm that my responses were seen.” I3  **Information about doctor:** “I would recommend that they do research about the doctor that they go to.” I4  **Link to other patients or support group**: “It is important to make friends with the people around you who are going through the same thing, because there will be someone who is a bit ahead of you who can guide you.” I3 |
|  |  | Valuable  *How can the platform add value to users?* | 6 | 5 | “I would actually do it more for the greater good to contribute to ongoing medical knowledge and learning.” I1  “I think I would still contribute my data if I knew it went for a good cause and if my doctor asked for it.” I2  “I actually think it may have more value for the person coming after me… Because the surgeon I will see again for my followup, but the anaesthetist you never see again, so it may help them to know how their patients are doing in the longer term after a procedure.” I3  “If it'll help someone with the same problem that I have, it's important to share it.” I5 |
|  | Potential Barriers | Confidentiality | 3 | 3 | “And no, personally I am not so concerned with the POPI act and confidentiality, as long as my data is de-identified.” I1  “I am very scared of random links and phishing scams. Even more so on email than on sms. If the sender is a person who is known to me, then I would do it, but if it looked phishy at all I would not click on the link.”I4 |
|  |  | Lack of Digital Literacy | 1 | 1 | “But then again, I do see that older patients struggle a lot and get anxious when they are asked to complete something online. So for me its easier, but it wont necessarily be easier for everyone.” I2 |
|  |  | Lack of interest | 2 | 2 | “After 6 months I think I will be motivated to, because at that stage I will still be on my postoperative journey, but I don’t know about 12 months, maybe after this yes, but if I think about other procedures like if someone had their appendix out, I’m not sure if they would want to still fill in questionnaires so far down the line.” I2 |
|  |  | Introduction | 1 | 1 | “I will be comfortable if maybe for an example if my doctor says fill up that thing because it's me and you. Doc, my doctor, treating doctor, then I'll be happy to. I can allow that to happen. I5 |

**Focus group 2: PSHR nonexposed (public healthcare sector).**

*Participants identified as “U” and a number according to the sequence in which they were interviewed.*

| **Main Theme** | **Subtheme** | **Code** | **Code Count** | **Case Count** | **Quote** |
| --- | --- | --- | --- | --- | --- |
| Patient journey  *How did the patient experience the time before and after their procedure?* | Information seeking behaviours  *What preparation did the patient undertake before their procedure?* | In person communication from doctor | 8 | 7 | “Yes, she did. She did give me another information. She was very clear. Oh she told me that I have to stay here without food the night. She explain to me about sleeping for the operation.” U1  “No, they just draw me pictures to show. It’s easier just to talk to them.” U9 |
|  |  | Information from other patients | 8 | 4 | “I'm not alone in the problem. Those who already went past this kind of question, I think they'll assist me. A group is something that we are sharing information, we are all learning.” U10 |
|  |  | Facility and doctor information | 8 | 4 | “And I did research about this facility. I did the research about the doctors. Um, I read on forums.” U5 |
|  |  | Video content | 6 | 4 | “I did quite a lot of extensive research, YouTube and TikTok is your friend.” U5  “Video, or reading also is fine.” U1 |
|  |  | Written media | 6 | 4 | “A pamphlet with pictures, even I can take them at home and be increasing my knowledge.” U10  “They also gave us some information leaflets and papers to go read at our own time.” U7 |
|  |  | Internet search | 4 | 3 | “I was checking how long it's going to be the operation. Okay. Yeah. And how was going to be the pain? How I was cut, a lot of it.” U1  “I go and check like, like the food I have to eat. And then the thing I didn't Google about it is the pills the most. But you know that if you want to go look, you can go, you can go find.” U3 |
|  |  | Avoidance | 3 | 3 | “So, I give up to an extent that I did not even want to stress myself about the Google information. Because others there are just making some speculations.” U10 |
|  |  | Information from family members | 1 | 1 | “My sister also had the operation, so I spoke to her a lot as well.” U7 |
|  | Emotional response  *What was the patient’s emotional response before their procedure?* | Emotional turmoil | 1 | 1 | “I was scared, but that time, the time they took me to the operation, I was relieved.” U1 |
|  |  | Frustration | 6 | 4 | “You know, actually they told me in three months, they have to remove the filter. I was coming for check every month from there. So when I come, when they supposed to admit me there was no bed. There was no bed for that. Five months I come for the treatment.” U3 |
|  |  | Hope and Faith | 2 | 2 | “I was not scared. You know me, I'm a strong woman. I can see that time I was taking things easier that everything will go well. You know, when you pray, and you know God, God is with you and he takes the burden with you.” U10 |
|  | Postoperative difficulties  *Difficulties that the patient had after their procedures.* | Positive experience | 4 | 3 | “The best thing is like, um, I get a good help the most than the other hospital. Here they check everything, take care of everything. And they keep people clean.” U3 |
|  |  | Mobilisation | 2 | 1 | “I am feeling better they can just remove the pipes then I can start walking and exercising, go to the park.” U2 |
|  |  | Physical discomfort | 2 | 2 | “But that was also the worst thing that I had the operation, because it was very painful! I didn’t expect it would be so painful! I wasn’t even friendly yesterday, but every day a bit better!” U7 |
|  | Interaction with healthcare providers  *Patient thoughts on the interaction with their health care team.* | Doctor / Nurse relationship to patient | 4 | 3 | “…what I felt was more these people are taking care of me. I was positive. I could see these other people (points to other patients in ward), they are getting more healthy.” U2  “At some other place I don't think they can do that operation. But here they explain they can do this operation. My doctors had explained to me everything.” U3  “I dont even think even in private they do such care. She was so supportive. She even gave me a massage. When you shout she's there.” U6 |
|  |  | Trust in the system | 10 | 6 | “No, I, I was fine. They are the one who work here, who knows what's going on with me and what we going to do.” U1  “No, you know actually, I didn't care because what I felt was more these people are taking care of me. I was positive. I could see these other people (points to other patients in ward), they are getting more healthy.” U2 |
|  | Advice to other patients  *Things the patient would have liked to know before going for surgery.* | Encourage to go ahead | 6 | 6 | “I would explain my journey the way it is, then they can come here and get that help because it's a better help than any other.” U3  “I will give them advice. I will say that they help you, they are good doctors.” U9 |
|  |  | Patience | 1 | 1 | “Mostly have patience, it's public. Okay. Some of the things we understand. Like when you're doing it private, it's very expensive, but here it is much better. But you must wait.” U6 |
|  |  | Physical preparation | 1 | 1 | “I give advice that they should stop drinking alcohol and stop cigarette. “U2 |
| PSHR Expectations  *What expectations would the patient have for a digital health tool like the PSHR?* | Use  *Using the product or application.* | Accessible  *Access to a device the patient will use the PSHR on*. | 24 | 10 | “Mmm, I also want to give this information. Just it is small on the phone. But I will try.” U1  “Anything that is easy for you is easy for me. But really Whatsapp is easiest.” U10  “We have emails, but we don’t use it so much.” U2  “It's easier to WhatsApp. I I'm always available on Whatsapp.” U5  “Phone is easiest, like I am booking my husband’s flights now, and doing it all on my phone.” U8  “The nurses took the phone and they give it the next day, because I arrived that really late time in the ward. They give it me in the morning.” U1 |
|  |  | Findable | 0 | 0 |  |
|  |  | Usable | 0 | 0 |  |
|  | Feel  *Emotional response users have when using the application.* | Desirable | 0 | 0 |  |
|  |  | Credible | 0 | 0 |  |
|  | Think  *Users’ cognitive engagement with an application.* | Useful  *What needs should the platform address?* | 21 | 7 | **Benchmarking**: “I mean, if they know what your baseline is, what my baseline is, how my life, my, my health is, you know, then they'll know how to proceed. With any procedure for that matter.” U5  **Communication**: “Because some people maybe they don't know how or like to explain to you how they feel, but as you are questioning them, they can talk and think and they can answer.” U3  **Efficiency**: “Because you are just sitting there waiting in any case, so this will help to spend the time wisely” U7  **Feedback**: “I would prefer feedback from the doctors saying based on my answers maybe I should change doing something.” U8 |
|  |  | Valuable  *How can the platform add value to users?* | 4 | 3 | **Personal connection**: “It shows you are not just a patient number one to 10.” U5  **Improved care**: “But when you check, keep on checking on your patient, it's good because if I feel something on me, I have to let you know. Then you'll ask me maybe then to come back at hospital. Then you can check that and sort it out.” U3 |
|  | Potential Barriers | Cost | 5 | 5 | “If I have data on it, it is not a problem. Only to have the data.” U1  “When I'm at home, I don't see that airtime. Because it's a cost of money. It's very expensive.” U3  “[Expensive data] makes it difficult to use the internet on the phone. There is wifi at my work and I use wifi.” U9 |
|  |  | Not comfortable with technology | 2 | 2 | “I'm completely technologically challenged. Okay. I had a laptop for two years and I didn't know how to switch it on.” U5  “The fancy phones, the internet, all that stuff, it’s not for me.” U4 |
